# Normative Values for the Short Physical Performance Battery (SPPB) and Their Association With Anthropometric Variables in Older Colombian Adults. The SABE Study, 2015

**DOI:** 10.3389/fmed.2020.00052

**Published:** 2020-02-20

**Authors:** Robinson Ramírez-Vélez, Miguel A. Pérez-Sousa, Luis C. Venegas-Sanabria, Carlos A. Cano-Gutierrez, Paula A. Hernández-Quiñonez, David Rincón-Pabón, Antonio García-Hermoso, Fabricio Zambom-Ferraresi, Mikel L. Sáez de Asteasu, Mikel Izquierdo

**Affiliations:** ^1^Navarrabiomed-Universidad Pública de Navarra (UPNA)-Complejo Hospitalario de Navarra (CHN), Instituto de Investigación Sanitaria de Navarra (IdiSNA), Pamplona, Spain; ^2^Faculty of Sport Sciences, University of Huelva, Huelva, Spain; ^3^Hospital Universitario San Ignacio – Aging Institute, Pontificia Universidad Javeriana, Bogotá, Colombia; ^4^GICAEDS Group, Faculty of Physical Culture, Sport and Recreation, Universidad Santo Tomás, Bogotá, Colombia; ^5^ZIPATEFI (Zona de Investigaciones de Posgrados, Terapia Respiratoria y Fisioterapia de Areandina), Fundación Universitaria del Área Andina, Pereira, Colombia; ^6^Laboratorio de Ciencias de la Actividad Física, el Deporte y la Salud, Universidad de Santiago de Chile, Santiago, Chile; ^7^CIBER of Frailty and Healthy Aging (CIBERFES), Instituto de Salud Carlos III, Madrid, Spain

**Keywords:** physical function, mobility, older people, reference values, latinos

## Abstract

**Background:** The short physical performance battery (SPPB) is a physical performance test of lower extremity function designed for non-disabled older adults. We aimed to establish reference values for community-dwelling Colombian adults aged 60 years or older in terms of (1) the total score; (2) the three subtest scores (walking speed, standing balance performance, and five times sit-to-stand test); and (3) the time to complete the five times sit-to-stand test, s and the walking speed test. Additionally, we sought to explore how much of the variance in the SPPB subtest scores could be explained by anthropometric variables (age, body mass, height, body mass index, and calf circumference).

**Methods:** Participants were men and women aged 60 years or older who participated in the Health and Well-being and Aging Survey in Colombia, 2015. A sample of 4,211 participants (57.3% women) completed the SPPB test, and their anthropometric variables were evaluated. Age-specific percentiles were calculated using the LMS method (3rd, 10th, 25th, 50th, 75th, 90th, and 97th percentiles).

**Results:** The mean SPPB total score for the entire sample was 8.73 (2.0) points. On average, the total SPPB score was 0.85 points greater in men than in women (*p* < 0.001). Significant sex differences were observed in all three age groups tested (60–69, 70–79, and 80+ years). In the full sample, our findings suggested that age, body mass, height, body mass index, and calf circumference are significant contributors to walking speed (*p* < 0.001) after controlling for confounding factors, including ethnicity, socioeconomic status, and urbanicity.

**Conclusions:** Percentile values are of interest to identify target populations for primary prevention and to estimate the proportion of high or low values for SPPB measures in community-dwelling Colombians aged at least 60 years.

## Introduction

Physical function (PF) is a key biomarker of disability, chronic disease, and mortality in older people (1). An assessment of PF provides a basis for an early evaluation of functional decline in older persons, whose PF ranges from vigorous to frail, and can guide geriatric treatment strategies (2). Furthermore, as an outcome measure, PF is a vital component of studies comparing groups or evaluating the effect of different interventions on functional status in older adults (3, 4).

We use the term “overall PF” to address the measurement of different physiological domains to generate an overall score. One of the most commonly used measures of PF is the short physical performance battery (SPPB), a well-established instrument used to assess lower extremity function that was developed to identify the onset of disability in older adults (5) and that is often used in community-dwelling adults (6), nursing home residents (7), and hospital settings (8). It is an objective tool for measuring lower extremity physical performance status. The SPPB is calculated from three components: (i) time to complete a 2.4, 3, or 4-m walk at the participant's usual pace, (ii) time to rise from a chair five times, and (iii) the ability to stand for up to 10 s with feet positioned in each of three ways (a side-by-side position, semi-tandem position, and tandem position) (9). The SPPB has been adopted in multiple observational studies, with higher scores indicating a higher level of PF and lower scores predicting adverse outcomes such as decreased mobility (10), falls (11), loss of independence in activities of daily living (ADLs) (12), hospitalization (8), longer hospital stays (13), nursing home admission (14), and all-cause mortality (15). Moreover, previous research has suggested that the SPPB can detect body composition changes (16, 17) and inflammation (18), and a total score ≤ 9 points can distinguish between vigorous and frail persons (19).

The importance of a multi-dimensional measurement of PF in older adults has been acknowledged in current primary care guidelines, and previous systematic reviews have concluded that the SPPB is a reliable and valid instrument for measuring lower limb strength in community-dwelling older people (6, 20, 21). Accordingly, it is considered a good endpoint for cross-cultural comparisons of physical performance in older individuals (19), and its validity has been confirmed in studies conducted in Brazil (22) and Colombia (23).

Reference or normative data provide an empirical context in which to explore PF and indicate the range of performance for a particular test in a particular population. In addition, physiological and anthropometric measures, such as body mass, height, and lower limb length, vary across ethnicities and are associated with physical performance. In this context, the use of reference data for a specific population is also essential for interpretation of the SPPB score (24). Thus, the optimal reference values for physical performance data must consider differences in sex, age, and variability in the community-dwelling settings of individuals.

Despite a growing body of research supporting the use of the SPPB test, there are relatively few large-scale normative reports in the literature (25). Indeed, there is an evident lack of up-to-date normative data on the SPPB assessing walking speed, standing balance performance and five times sit-to-stand test. The latest published SPPB data on community-dwelling older people are from a Norwegian regional study of 7,474 adults and older adults (40–85 years) (26) and from a regional study in Spain of 593 persons ≥70 years of age (27). At present, there are no published reference values for the SPPB (in terms of total score) based on a large sample of individuals aged 60+ years in the Latin American population. The distinctive diet, habitat, health status, race, and geographical location of general Colombian populations have significant impacts on anthropometric and physical performance data.

Given the above, we aimed to establish reference values, stratified by sex and age, for community-dwelling Colombian adults aged 60–95 years in terms of (1) the SPPB total score, as recommended by Steffen et al. (25) and (2) the scores of the three subtests (walking speed, standing balance performance and five repetitions of the sit-to-stand test). Additionally, we sought to explore how much of the variance in SPPB subtest scores could be explained by anthropometric variables [age, body mass, height, and body mass index (BMI)] affecting these measures.

## Materials and Methods

### Design, Setting, and Participants

SABE Colombia Survey was conducted in 2015 by the Epidemiological Office of the Ministry of Health and Social Protection of Colombia (https://www.minsalud.gov.co/). Participants comprised men and women aged 60+ years, residing in urban and rural households in all regions of Colombia, who were non-institutionalized and who were Spanish speakers. The sample was probabilistic, clustered, stratified/multistage by urban and rural areas, and the stages (municipalities, segments, housing, and homes). Written informed consent was obtained from older adults, and the survey was reviewed and approved by the institutional review boards of the University of Caldas (ID protocol CBCS-021-14) and the University of Valle (ID protocol 09-014 and O11-015); the secondary analysis was approved by Pontificia Universidad Javeriana (ID protocol 20/2017-2017/180, FM-CIE-0459-17) in accordance with the Declaration of Helsinki of the World Medical Association and with Resolution 8430 (1993) of the Ministry of Health of Colombia. The technical details of the SABE survey have been published previously (28).

The study constituted 99% of the population and calculation of the sample was carried out taking into account the regional disaggregation and the forced inclusion of the four large cities (3,500 individuals per city by accumulating the sample values for the sub region, region, and country). According to the National Administrative Department of Statistics commonly referred to as DANE, 4,964,793 older adults living in Colombia in the 2013. Thus, parameters used sample size calculation were (0.03) of minimum expected proportion (1.2), of design effect (0.05), of relative standard error, and ~20% of non-response percentage. According to the previous description, 30,691 surveys were estimated at the national level, 23,162 in the urban area (75.5%) and 7,529 in the rural area (1,908 in populated centers and 5,621 in dispersed rural areas). A total of 6,530 segments, 4,928 urban and 1,602 rural, were planned to obtain the surveys, with an expected average of 4.7 adults per segment.

Functionality tests in a subsample of older adults including grip strength, walking speed, balance, and time to get up from a chair measure. For this subsample, the calculation of the sample size was carried out taking into account national representation, by randomly selecting one for every two individuals of the survey, obtaining a sample of 6,161 people +60 years of age. The estimate took into account an expected proportion of ~6%, a maximum error of 6% and a non-response percentage of 20%. Visual inspection of the data using boxplots revealed ~10% outliers (determined using the interquartile rule) for both walking speed, balance tests, and anthropometric variables. Additionally, we included individuals who completed the SPPB with non-missing values for all subtests. Thus, our final study population comprised 4,211 participants (57.3% women).

### Study Variables

A structured interview was administered to obtain socio-demographic data, which included age groups (60–69, 70–79, and 80+), gender (men and women), ethnic group (indigenous, black “mulato” or Afro-Colombian, white and others), and socioeconomic status (level I–II: low; level II–III: middle, and level V–VI: high), as well as anthropometric and SPPB test, were measured and collected according to the standard procedures previously published in “The SABE Technical Report” (28). Height (SECA 213®, Hamburg, Germany) to the nearest 0.1 cm and body mass (Kendall graduated platform scale) with a precision of 0.1 kg, were measured with the subject wearing light indoor clothing (28, 29). BMI was calculated using the formula BMI = weight (kg)/height (m^2^). Calf circumference (CC) was measured to the nearest 0.1 cm in the standing position using a non-elastic tape measure.

The SPPB was administered using standardized methodologies for the instructions, positioning, and scoring by trained staff (23). To assess usual walking speed (meters/second), the participants were asked to walk 3 m at their regular pace two times, from standing position. The standing balance tests included side-by-side, semi-tandem, and full-tandem standing, and the participants were timed until they moved, or 10 s had elapsed. To assess the five times sit-to-stand test, the participants were asked to perform five chair stands as quickly as possible. Time (in seconds) was registered with a stopwatch with a resolution of 0.01 s. Scores of 0–4 points (maximum performance) were assigned for each subtest based on timed quartiles that were established previously in a large population study, according to the standard procedures previously published by Guralnik et al. (5) Scores results derived from the three timed physical performance was divide as 0–3, 4–6, 7–9, and 10–12 subgroups.

### Statistical Methods

All data were analyzed using SPSS version 24.0 for Windows (SPSS, Chicago, IL, USA) and MedCalc Statistical Software version 18.2 for Windows (MedCalc Software BVBA, Ostend, Belgium). The normality of variables was verified with the Kolmogorov-Smirnov test and probability plots. Crude mean values and standard deviations (SD) stratified by sex and age were first determined. Student's *t*-test was applied to identify significant differences in continuous variables, and a chi-squared test was used for categorical variables. In addition, the effect sizes (Cohen's *d*) were calculated to evaluate differences in continuous variables. The effect size was interpreted by using trivial (<0.20), small (0.20– <0.50), moderate (0.50– <0.80), and large (≥0.80) values (30). To generate sex-specific and age-specific normative centiles (3rd, 10th, 25th, 50th, 75th, 90th, and 97th), we applied the Lambda Mu Sigma (LMS) method (31) using LMS chartmaker Pro (v2.43, The Institute of Child Health, London, UK). The LMS method fits smooth centile curves to reference data by summarizing the changing distribution of three sex-specific and age-group date representing the skewness (L; expressed as a Box-Cox power), the median (M) and the CV (S) (32). The LMS method was run separately for men and women. A regression analysis (standardized regression coefficient) was performed to analyze how much of the variance in continuous scores of the SPPB subtests (walking speed and five times sit-to-stand test) can be explained by anthropometric variables (age, body mass, height, BMI, and CC) as crude model analysis. Second model was adjusted for ethnicity, socioeconomic status and urbanicity. The covariates included in the adjusted analyses were based on conceptual model according to the literature. As CC has been more closely related to nutritional status and physical performance (33), we chose it as the main indicator for surrogate marker of muscle mass for diagnosing sarcopenia in the standard multiple regression analysis. A statistical significance value of probability was set to *p* < 0.05.

## Results

General characteristics (sex, age, height, weight, BMI, socioeconomic status, urbanicity, and ethnic group) are shown separately for men and women in Table 1. The mean (SD) age of the total sample (4,211 participants, 57.3% women) was 69.0 (6.8), range 60–95 years. The mean total SPPB score for men and women was 9.2 (2.0), range, 2–12 points, and 8.4 (2.0), range, 2–12 points, respectively. In both men and women, the distribution of SPPB scores showed a decreased function with increased age in all three age groups.

**Table 1:** General characteristics and SPPB score distribution according to sex and age, *n* = 4,211.

**Age groups (years)**	**60–69**	**70–79**	**80+**	**Total**
**Men**, ***n***	985	603	207	1,795
Mean age (SD)	64.4 (2.8)	73.8 (2.8)	83.4 (3.2)	69.7 (7.1)
Mean height, m (SD)	1.64 (0.07)	1.62 (0.10)	1.61 (0.07)	1.63 (0.08)
Mean body mass, kg (SD)	69.5 (12.9)	67.4 (11.7)	63.5 (10.5)	68.1 (12.4)
Mean BMI, kg/m^2^ (SD)	26.4 (4.2)	26.1 (3.9)	25.3 (3.5)	26.2 (4.1)
Mean calf circumference, cm (SD)	35.1 (3.4)	34.5 (3.3)	33.4 (3.0)	34.7 (3.4)
**SOCIOECONOMIC STATUS**				
Level I–II (low)	79.7	76.9	80.6	78.9
Level II–III (middle)	19.7	22.5	18.8	20.5
Level V–VI (high)	0.6	0.5	0.5	0.6
**URBANICITY, %**				
Urban	75.0	71.6	73.4	73.7
Rural	25.0	28.4	26.6	26.3
**ETHNIC GROUP (SELF-REPORT), %**				
Indigenous	8.8	10.1	3.5	8.7
Black “mulato” or Afro-Colombian	10.3	11.4	12.5	10.9
White	28.2	28.3	33.3	28.7
Others^*^	52.8	50.2	50.7	51.7
**SPPB SCORE, %**				
0–3	0.2	0.8	3.9	0.8
4–6	3.5	9.5	29.0	8.4
7–9	34.6	49.3	47.3	41.0
10–12	61.7	40.5	19.8	49.7
Mean SPPB score (SD)	9.8 (1.7)	8.9 (1.9)	7.5 (2.2)	9.2 (2.0)
**Women**, ***n***	1,420	781	215	2,416
Mean age (SD)	64.2 (2.8)	73.9 (2.8)	83.1 (3.0)	69.1 (6.8)
Mean height, m (SD)	1.52 (0.07)	1.50 (0.08)	1.49 (0.06)	1.51 (0.08)
Mean weight, kg (SD)	64.5 (12.9)	61.1 (12.2)	56.0 (11.1)	62.7 (12.8)
Mean BMI, kg/m^2^ (SD)	28.7 (5.3)	28.1 (5.2)	25.9 (4.8)	28.3 (5.3)
Mean calf circumference, cm (SD)	35.1 (3.8)	34.0 (3.9)	32.8 (3.8)	34.6 (3.9)
**SOCIOECONOMIC STATUS, %**				
Level I–II (low)	75.9	73.3	67.4	74.3
Level II–III (middle)	23	25.5	31.7	24.5
Level V–VI (high)	1.1	1.2	0.9	1.1
**URBANICITY, %**				
Urban	80.1	79.6	83.3	80.2
Rural	19.9	20.4	16.7	19.8
**ETHNIC GROUP (SELF-REPORT), %**				
Indigenous	6.0	4.2	2.2	5.2
Black “mulato” or Afro-Colombian	9.1	7.9	7.2	8.6
White	32.2	33.6	48.6	33.7
Others^*^	52.7	54.2	42.0	52.5
**SPPB SCORE, %**				
0–3	0.5	1.9	0.9	1.0
4–6	7.5	20.0	44.2	14.8
7–9	52.7	58.4	47.9	54.1
10–12	39.4	19.7	7.0	30.1
Mean SPPB score (SD)	8.9 (1.8)	7.9 (2.0)	6.7 (1.8)	8.4 (2.0)

Data are presented as mean ± SD or % (percentage) of participants. SD, standard deviation; BMI, Body mass index.

* *Others (mestizo, gypsy/ROM, etc.)*.

The distribution of scores for each of the subtests is shown in Table 2. The mean (SD) walking speed, standing balance performance and five sit-to-stand test scores for the total sample (*n* = 4,211) were 2.8 (0.9), 3.6 (0.8), and 2.2 (1.1), respectively. When analyzing the sexes separately, the male 60–69-year age group had significantly higher scores than other male age groups for walking speed, 3.3 (0.8) *p* < 0.001, standing balance performance, 3.8 (0.6) *p* < 0.001, and five times sit-to-stand test, 2.7 (1.1) *p* < 0.001, whereas the female group 60–69 years had significantly higher scores than other female age groups for walking speed, 2.9 (0.9) *p* < 0.001, standing balance performance, 3.7 (0.8) *p* < 0.001, and five times sit-to-stand test, 2.3 (1.0) *p* < 0.001.

**Table 2:** Distribution of SPPB subtest scores according to sex and age, *n* = 4,211.

**Sex, age (years), and SPPB subtest**	**Mean score (SD)**	**Subtest score**				
		**0**	**1**	**2**	**3**	**4**
**MEN**						
**60–69**, ***n*** **=** **985**						
Walking speed	3.3 (0.8)	–	31 (3.1)	160 (16.2)	307 (31.2)	487 (49.4)
Standing balance performance	3.8 (0.6)	7 (0.7)	12 (1.2)	27 (2.7)	58 (5.9)	881 (89.4)
Five times sit-to-stand test	2.7 (1.1)	–	182 (18.5)	229 (23.2)	281 (28.5)	293 (29.7)
**70–79**, ***n*** **=** **603**						
Walking speed	3.0 (0.9)	–	23 (3.8)	167 (27.7)	199 (33.0)	214 (35.5)
Standing balance performance	3.6 (0.8)	7 (1.2)	19 (3.2)	38 (6.3)	53 (8.8)	486 (80.6)
Five times sit-to-stand test	2.2 (1.1)	–	191 (31.7)	176 (29.2)	132 (21.9)	104 (17.2)
**80+**, ***n*** **=** **207**						
Walking speed	2.4 (1.0)	–	38 (18.4)	77 (37.2)	56 (27.1)	36 (17.4)
Standing balance performance	3.2 (1.1)	4 (1.9)	17 (8.2)	30 (14.5)	32 (15.5)	124 (59.9)
Five times sit-to-stand test	1.9 (1.0)	–	106 (51.2)	44 (21.3)	39 (18.8)	18 (8.7)
**All ages**, ***n*** **=** **1,795**						
Walking speed	3.0 (0.9)	–	92 (5.1)	404 (22.5)	562 (31.3)	737 (41.1)
Standing balance performance	3.6 (0.7)	18 (1.0)	48 (2.7)	95 (5.3)	143 (8.0)	1,491 (83.1)
Five times sit-to-stand test	2.4 (1.1)	–	479 (26.7)	449 (25.0)	452 (25.2)	415 (23.1)
**WOMEN**						
**60–69**, ***n*** **=** **1,420**						
Walking speed	2.9 (0.9)	–	67 (4.7)	404 (28.5)	511 (36.0)	438 (30.8)
Standing balance performance	3.7 (0.8)	18 (1.3)	39 (2.7)	69 (4.9)	107 (7.5)	1,187 (83.6)
Five times sit-to-stand test	2.3 (1.1)	–	439 (30.9)	398 (28.0)	359 (25.3)	224 (15.8)
**70–79**, ***n*** **=** **781**						
Walking speed	2.6 (1.0)	–	100 (12.8)	287 (36.7)	235 (30.1)	159 (20.4)
Standing balance performance	3.4 (1.1)	20 (2.6)	48 (6.1)	73 (9.3)	101 (12.9)	539 (69.0)
Five times sit-to-stand test	1.9 (1.0)	–	369 (47.2)	199 (25.5)	127 (16.3)	86 (11.0)
**80+**, ***n*** **=** **215**						
Walking speed	2.1 (0.8)	–	58 (27.0)	99 (46.0)	47 (21.9)	11 (5.1)
Standing balance performance	3.0 (1.1)	4 (1.9)	17 (7.9)	61 (28.4)	29 (13.5)	104 (48.4)
Five times sit-to-stand test	1.6 (0.9)	–	130 (60.5)	47 (21.9)	22 (10.2)	16 (7.4)
**All ages**, ***n*** **=** **2,416**						
Walking speed	2.7 (0.9)	–	225 (9.3)	790 (32.7)	793 (32.8)	608 (25.2)
Standing balance performance	3.5 (0.9)	42 (1.7)	104 (4.3)	203 (8.4)	237 (9.8)	1,830 (75.7)
Five times sit-to-stand test	2.0 (1.0)	–	938 (38.8)	644 (26.7)	508 (21.0)	326 (13.5)
**TOTAL SAMPLE**						
**60–69**, ***n*** **=** **2,405**						
Walking speed	3.1 (0.9)	–	98 (4.1)	564 (23.5)	818 (34.0)	925 (38.5)
Standing balance performance	3.7 (0.7)	25 (1.0)	51 (2.1)	96 (4.0)	165 (6.9)	2,068 (86.0)
Five times sit-to-stand test	2.4 (1.1)	–	621 (25.8)	627 (26.1)	640 (26.6)	517 (21.5)
**70–79**, ***n*** **=** **1,384**						
Walking speed	2.8 (0.9)	–	123 (8.9)	454 (32.8)	434 (31.4)	373 (27.0)
Standing balance performance	3.5 (1.0)	27 (2.0)	67 (4.8)	111 (8.0)	154 (11.1)	1,025 (74.1)
Five times sit-to-stand test	2.1 (1.1)	–	560 (40.5)	375 (27.1)	259 (18.7)	190 (13.7)
**80+**, ***n*** **=** **422**						
Walking speed	2.2 (0.9)	–	96 (22.7)	176 (41.7)	103 (24.4)	47 (11.1)
Standing balance performance	3.1 (1.1)	8 (1.9)	34 (8.1)	91 (21.6)	61 (14.5)	228 (54.0)
Five times sit-to-stand test	1.7 (1.0)	–	236 (55.9)	91 (21.6)	61 (14.5)	34 (8.1)
**All ages**, ***n*** **=** **4,211**						
Walking speed	2.8 (0.9)	–	317 (7.5)	1,194 (28.4)	1,355 (32.2)	1,345 (31.9)
Standing balance performance	3.6 (0.8)	60 (1.4)	152 (3.6)	298 (7.1)	380 (9.0)	3,321 (78.9)
Five times sit-to-stand test	2.2 (1.1)	–	1,417 (33.6)	1,093 (26.0)	960 (22.8)	741 (17.6)

*SPPB subtest scores ranged 0–4. Data are presented as number of participants (%). SD, standard deviation*.

The mean (SD) walking speed and five times sit-to-stand test scores, as well as the 3rd−97th percentiles and LMS values, are shown in Tables 3–5. For the whole sample, walking speed was significantly greater in men than in women (*diff* = 1.0 m/s, *d* = 0.391, *p* < 0.001). Furthermore, the decline in walking speed with age was similar across sexes until the age of 80+ years; starting at this age, the decline in women was greater than that in men (*diff* = 0.08 m/s, *d* = 0.467, *p* < 0.001). Performance in the five sit-to-stand tests was different between men and women in the 70–79-year group (*diff* = 1.37 s, *d* = 0.319, *p* < 0.001) and the 80+-year group (*diff* = 0.03 s, *d* = 0.208, *p* < 0.001), with women performing significantly worse than men from 60–69 years of age onward (*diff* = 1.42 s, *d* = 0.402, *p* < 0.001).

**Table 3:** Distribution of SPPB subtest scores (walking speed, and five times sit-to-stand test) by sex and age.

**Age (years)**	**Men**		**Women**		**Total**		*P*-value^*^ (Cohen's *d*)
	***N***	**Mean (SD)**	***N***	**Mean (SD)**	***N***	**Mean (SD)**	
**WALKING SPEED, M/S**							
60–69	985	0.88 (0.29)	1,420	0.77 (0.24)	2,405	0.82 (0.26)	<0.001 (0.419)
70–79	603	0.79 (0.23)	781	0.69 (0.22)	1,384	0.73 (0.23)	<0.001 (0.462)
80+	207	0.66 (0.21)	215	0.58 (0.15)	422	0.62 (0.19)	<0.001 (0.467)
All ages	1,795	0.83 (0.27)	2,416	0.73 (0.23)	4,211	0.77 (0.26)	<0.001 (0.391)
**FIVE TIMES SIT-TO-STAND TEST, S**							
60–69	985	11.93 (5.10)	1,420	13.35 (5.71)	2,405	12.77 (5.51)	<0.001 (0.407)
70–79	603	13.59 (5.51)	781	14.96 (6.09)	1,384	14.36 (5.88)	<0.001 (0.319)
80+	207	15.97 (6.14)	215	16.00 (7.02)	422	15.98 (6.58)	0.033 (0.208)
All ages	1,795	12.95 (5.52)	2,416	14.10 (6.03)	4,211	13.60 (5.84)	<0.001 (0.327)

* *Significant difference by sex was analyzed by t-test. SD, standard deviation*.

Values of walking speed (3rd−97th percentile) changed by age for both sexes (Table 4), and the same pattern was found in the five repetitions of the sit-to-stand test (Table 5). Table 6 summarizes the corresponding percentiles and LMS values for the total SPPB score for the groups 60–69, 70–79, and 80+ years for the total sample and by sex. Thus, Colombian older people can also be classified into SPPB scores such as very low (SPPB <3rd percentile), low (3rd ≤ SPPB <25th percentile), medium (25th ≤ SPPB <75th percentile), high (75th ≤ SPPB <97th percentile), and very high (SPPB ≥ 97th percentile).

**Table 4:** Smooth centile scores and LMS values for the SPPB walking speed (in m/s) test by age and sex.

**Age (years)**	***N***	**L**	**S**	**3rd**	**10th**	**25th**	**50th (M)**	**75th**	**90th**	**97th**
**MEN**										
60–69	985	−0.03	0.31	0.46	0.56	0.68	0.84	1.03	1.27	1.56
70–79	603	−0.10	0.30	0.42	0.51	0.62	0.75	0.92	1.13	1.40
80+	207	−0.17	0.29	0.36	0.43	0.52	0.63	0.76	0.93	1.15
**WOMEN**										
60–69	1,420	−0.17	0.29	0.42	0.50	0.61	0.74	0.90	1.10	1.37
70–79	781	−0.24	0.29	0.38	0.45	0.54	0.65	0.79	0.97	1.20
80+	215	−0.31	0.28	0.33	0.39	0.46	0.55	0.67	0.82	1.01
**TOTAL**										
60–69	2,405	−0.13	0.31	0.43	0.52	0.63	0.78	0.95	1.18	1.47
70–79	1,384	−0.18	0.30	0.39	0.47	0.57	0.69	0.85	1.05	1.30
80+	422	−0.24	0.29	0.34	0.40	0.48	0.59	0.72	0.88	1.10

*LMS, lambda, mu, and sigma; SD, standard deviation. Degree of freedom (L = 2, M = 3, and L = 2)*.

**Table 5:** Smooth centile scores and LMS values for the SPPB five times sit-to-stand test (in seconds) by age and sex.

**Age (years)**	**N**	**L**	**S**	**3rd**	**10th**	**25th**	**50th (M)**	**75th**	**90th**	**97th**
**MEN**										
60–69	985	0.43	0.30	6.33	8.16	10.25	12.62	15.27	18.21	21.45
70–79	603	0.36	0.29	7.39	9.39	11.71	14.35	17.35	20.72	24.48
80+	207	0.30	0.29	8.47	10.64	13.17	16.10	19.46	23.28	27.60
**WOMEN**										
60–69	1,420	0.88	0.29	6.42	9.03	11.74	14.53	17.38	20.30	23.26
70–79	781	0.76	0.29	7.51	10.16	12.99	15.97	19.11	22.37	25.75
80+	215	0.64	0.28	8.63	11.31	14.25	17.42	20.83	24.45	28.27
**TOTAL**										
60–69	2,405	0.56	0.30	6.55	8.66	11.02	13.63	16.48	19.57	22.88
70–79	1,384	0.54	0.30	7.46	9.77	12.35	15.21	18.35	21.75	25.41
80+	422	0.52	0.29	8.40	10.89	13.70	16.81	20.22	23.92	27.93

*LMS, lambda, mu, and sigma; SD, standard deviation. Degree of freedom (L = 2, M = 3, and L = 2)*.

**Table 6:** Smooth centile scores and LMS values for total SPPB score by age and sex.

**Age (years)**	**N**	**L**	**S**	**3rd**	**10th**	**25th**	**50th (M)**	**75th**	**90th**	**97th**
**MEN**										
60–69	985	2.38	0.15	6	8	9	10	11	12	12
70–79	603	1.78	0.21	4	6	8	9	10	11	12
80+	207	1.18	0.26	3	5	6	8	9	10	12
**WOMEN**										
60–69	1,420	1.67	0.19	5	6	8	9	10	11	12
70–79	781	1.19	0.24	4	5	7	8	9	10	12
80+	215	0.71	0.29	3	4	5	7	8	9	11
**TOTAL**										
60–69	2,405	1.88	0.18	5	7	8	9	10	11	12
70–79	1,384	1.37	0.23	4	6	7	8	10	11	12
80+	422	0.86	0.29	3	4	6	7	8	10	11

*LMS, lambda, mu, and sigma; SD, standard deviation. Degree of freedom (L = 2, M = 3, and L = 2)*.

Age and BMI were found to be inversely associated with walking speed in both men and women (β = −0.293 to −0.041; and β = −0.298 to −0.040, respectively, *p* < 0.001) and in the full sample (β = −0.280 to −0.077, *p* < 0.001), while walking speed was directly associated with height (men: β = 0.101, women: β = 0.097, and full sample: β = 0.189, *p* < 0.001) and CC (men: β = 0.071, women: β = 0.062, and full sample: β = 0.067, *p* < 0.01). Additionally, higher five- repetition sit-to-stand test scores were related to lower values of body mass (β = −0.032, *p* < 0.001) and height (β = −0.070, *p* < 0.001) in males. In females, the largest change was observed in the five sit-to-stand test scores with each one-year increase in age (β = 0.182, *p* < 0.001). Additionally, the five times sit-to-stand test scores decreased by −0.021 s per kg increase in body mass in females (*p* < 0.001). The equivalent for the full sample was found for the five repetitions of the sit-to -stand test (β = −0.020) in body mass and height (β = −0.096), *p* < 0.001. In both the sex and full-sample analyses, these associations persisted even after further adjustment (ethnicity, socioeconomic status, and urbanicity) for age (men: β = −0.260, women: β = −0.270, and full sample: β = −0.248, *p* < 0.001), height (men: β = 0.091, women: β = 0.086, and full sample: β = 0.186, *p* < 0.001), BMI (only in women: β = −0.049, and full sample: β = −0.082, *p* < 0.05), CC (only in men: β = 0.063, and full sample: β = 0.052, *p* < 0.05), and walking speed (Table 7).

**Table 7:** SPPB subtests (walking speed and five times sit-to-stand test) in relation to anthropometric variables.

**Anthropometric variables**	**Men**		**Women**		**Total**	
	**Beta (β)^a^**	***P*-value**	**Beta (β)^a^**	***P*-value**	**Beta (β)^a^**	***P*-value**
**UNADJUSTED MODEL**						
**Age, years**						
Walking speed, m/s	−0.293	<0.001	−0.298	<0.001	−0.280	<0.001
Five times sit-to-stand test, s	0.231	<0.001	0.182	<0.001	0.159	<0.001
**Body mass, kg**						
Walking speed, m/s	0.036	<0.001	0.020	<0.001	0.066	<0.001
Five times sit-to-stand test, s	−0.032	<0.001	−0.021	<0.001	−0.020	<0.001
**Height, cm**						
Walking speed, m/s	0.101	<0.001	0.097	<0.001	0.189	<0.001
Five times sit-to-stand test, s	−0.070	0.004	−0.024	0.252	−0.096	<0.001
**BMI, kg/m**^**2**^						
Walking speed, m/s	−0.041	<0.001	−0.040	<0.001	−0.077	<0.001
Five times sit-to-stand test, s	−0.001	0.952	0.036	0.090	0.044	0.006
**Calf circumference, cm**						
Walking speed, m/s	0.071	0.003	0.062	0.002	0.067	<0.001
Five times sit-to-stand test, s	−0.022	0.360	−0.018	0.369	−0.021	0.167
**ADJUSTED MODEL^b^**						
**Age, years**						
Walking speed, m/s	−0.260	<0.001	−0.270	<0.001	−0.248	<0.001
Five times sit-to-stand test, s	0.207	<0.001	0.140	<0.001	0.163	<0.001
**Body mass, kg**						
Walking speed, m/s	0.030	0.227	−0.008	0.717	0.051	0.002
Five times sit-to-stand test, s	0.004	0.889	−0.059	0.009	0.006	0.733
**Height, cm**						
Walking speed, m/s	0.091	<0.001	0.086	<0.001	0.186	<0.001
Five times sit-to-stand test, s	−0.031	0.223	−0.024	0.283	−0.078	<0.001
**BMI, kg/m**^**2**^						
Walking speed, m/s	−0.039	0.124	−0.049	0.028	−0.082	<0.001
Five times sit-to-stand test, s	0.015	0.566	−0.064	0.006	0.058	0.001
**Calf circumference, cm**						
Walking speed, m/s	0.063	0.013	0.037	0.094	0.052	0.002
Five times sit-to-stand test, s	−0.011	0.671	−0.004	0.871	−0.008	0.623

a Standardized regression coefficient;

b *analysis was adjusted by ethnicity, socioeconomic status, and urbanicity*.

## Discussion

The present study provides epidemiologic data on age and sex-specific SPPB total scores, as well as for the three subtests included in the SPPB by percentiles. To the best of our knowledge, this study is the first to examine the association between anthropometric variables and PF in a representative sample of community-dwelling Colombian older adults. Furthermore, we confirm that the main decline in PF occurs in the mid-sixties, with a slightly earlier decline in women than in men, which is consistent with a previous study (9).

In a previous meta-analysis, Bohannon et al. (34) reported a clear effect of age on walking speed, stratified by sex and age group (in 10-year intervals), which corresponds quite well to the present results. The present data on walking speed in men and women at different ages are in line with the reference values for standardized tests of walking speed by Thaweewannakij et al. (9) and Cabrero-García et al. (27) the Tromsø Study by Bergland et al. (26) and Guralnik et al. (5), see Table 8. Our findings are consistent with previous studies (5, 9, 26, 27) indicating that women might experience a decline in walking speed, whereas males might experience a parallel decline in “overall physical performance.” Overall walking speed varied from 0.58 to 0.88 m/s, according to our findings, even though the walking distances differed between studies. We observed moderate but significant differences (13%, *d* = 0.419; *p* < 0.0001) between the sexes for the 60–69-year age group, which increased to 25% (*diff* = 0.2 m/s) in older adults (80 years). In those participants aged 80 years or older, the magnitude of between-sex differences (12%, *d* = 1.06; *p* < 0.0001) was greater than that of subjects from Norway (3%) and Thailand (9%). It is crucial to consider the distance over which the walking speed is calculated when making comparisons. For example, if we use a cut-off point of <1 m/s, which has been used for the 6-m test (35), 85% of the sample will be at risk of having a health-related adverse event, instead of 38%, if using a cut-off point of <0.6 m/s on the 4-m test (23). Specifically, a decrease in gait speed of 0.1 m/s has been associated with a 10% decrease in the ability to perform instrumental ADLs (36).

**Table 8:** Comparison of the mean values of walking speed and five times sit-to-stand test from cited studies.

	**Present study** **Colombia**	**The Tromsø Study** **Norway**		**Thaweewannakij et al.** **Thailand**		**Cabrero-García et al.** **Spain**		**Guralnik et al.** **United States**	
**Men**	**Mean (SD)^a^**	**Men**	**Mean (SD)^b^**	**Men**	**Mean (SD)^a^**	**Men**	**Mean (SD)^b^**	**Men**	**Mean**
**WALKING SPEED (M/S)**									
60–69	0.88 (0.29)	60–64	1.21 (0.2)	60–69	1.16 (0.2)	70–75	0.9 (0.2)	–	–
		65–69	1.18 (0.2)						
70–79	0.79 (0.23)	70–75	1.12 (0.2)	70–79	1.09 (0.2)	76–80	0.8 (0.2)	–	–
		75–79	1.03 (0.2)						
80+	0.66 (0.21)	80+	0.97 (0.2)	80+	0.97 (0.2)	80+	0.7 (0.2)	–	–
**Women**		**Women**		**Women**		**Women**	**Mean (SD)^b^**	**Women**	**Mean**
60–69	0.77 (0.24)	60–64	1.20 (0.2)	60–69	1.08 (0.1)	70–75	0.8 (0.2)	–	–
		65–69	1.13 (0.2)						
70–79	0.69 (0.22)	70–75	1.08 (0.2)	70–79	0.99 (0.1)	76–80	0.7 (0.2)	–	–
		75–79	1.00 (0.2)						
80+	0.58 (0.15)	80+	0.94 (0.2)	80+	0.88 (0.1)	80+	0.6 (0.2)	–	–
**Men**	**Mean (SD)**	**Men**	**Mean (SD)**	**Men**	**Mean (SD)**	**Men**	**Mean (SD)**	**Men**	**Mean**
**FIVE TIMES SIT-TO STAND-TEST (S)**									
60–69	11.93 (5.10)	60–64	8.7 (2.4)	60–69	12.9 (3.2)	70–75	–	71+	13.7
		65–69	9.2 (2.8)						
70–79	13.59 (5.51)	70–75	9.7 (2.7)	70–79	13.5 (3.5)	76–80	–	71–79	13.2
		75–79	10.7 (2.9)						
80+	15.97 (6.14)	80+	11.9 (3.8)	80+	14.2 (3.4)	80+	–	80+	15.0
**Women**		**Women**		**Women**		**Women**	**Mean (SD)**	**Women**	**Mean**
60–69	13.35 (5.71)	60–64	9.4 (2.7)	60–69	13.2 (2.8)	70–75	–	71+	14.9
		65–69	10.5 (3.2)						
70–79	14.96 (6.09)	70–75	11.3 (3.3)	70–79	14.7 (3.6)	76–80	–	71–79	14.4
		75–79	11.7 (3.2)						
80+	16.00 (7.02)	80+	12.6 (4.1)	80+	17.1 (4.6)	80+	–	80+	16.1

a Walk 3 m;

b *walk 4 m; (–) Not informed*.

Considering the five repetitions of the sit-to-stand test, the time varied from 11.9 to 15.9 s and from 13.3 to 16.0 s among age groups for men and women, respectively. By sex group (aged 60–69 years), we observed medium but significant differences (*diff* = 1.42 s; *d* = 0.407; *p* < 0.001), which was similar to findings reported in all age groups (*diff* = 1.15 s; *d* = 0.327; *p* < 0.001). In the study by Thaweewannakij et al. (9) and in the original SPPB study by Guralnik et al. (5) the times ranged from 13.5 to 14.9 s in the 70–79-year age group to 16.1–17.1 s in the 80 or older age group in females, whereas males displayed higher scores in the 70–79-year age group (12.9–13.7 s) and the 80 years or older age group (14.2–15.0 s). The discrepancy between the results of these studies and our findings could be related to the position of the arms and the seat height. Furthermore, the seat height was not individually adjusted for each participant. Standing from an inappropriate seat height may affect the outcomes of the five repetitions of the sit-to-stand test (37).

The findings of the present study suggest that age, body mass, height BMI, and CC are all significant contributors to the functional ability of the participants, independent of sex (*p* < 0.001). For example, an increase in age and BMI was associated with lower test scores for walking speed, and walking speed was directly associated with height values. Additionally, higher five times sit-to-stand test scores were related to lower values for body mass and height in males, while for females, the largest change in the five sit-to-stand test scores was related to age and body mass. Several studies have reported that a higher BMI is associated with reduced levels of PF, and previous cross-sectional studies have shown that overweight and obesity are associated with walking speed or timed up-and-go test performance (38, 39). These findings are consistent because PF has been shown to decrease with increasing age (40–42), in part due to the decline in muscle strength from 40 to 50 years of age (43). The age-related declines observed in our study in neuromuscular endurance and explosive power are supported by previous findings (29, 33). In this regard, Bassey et al. (44) reported that muscle strength declines annually by ~1–1.5% between 50 and 60 years of age and by 3% after 60 years of age. The present study, however, is cross-sectional and cannot be used to determine cause and effect relationships between anthropometric variables and physical performance or the cause of differences in scores of the various PF tests observed between age- and sex-specific groups. Nevertheless, the health status, age, sex, and controlled factors, including daily lifestyle and levels of physical exercise, of the participants may be independent factors for determining levels of physical performance in older adults (45).

**Figure 1:**
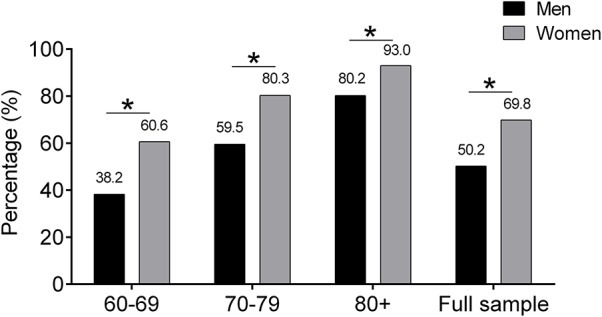
Share of individuals with a frailty phenotype using ≤ 9 cut-off point. The graph illustrates the rising percentage of men and women with ≤ 9 cut-off point by SPPB score that lie frailty phenotype.

Finally, the mean total SPPB scores of the male and female samples were 9.2 (2.0), range 2–12 points, and 8.4 (2.0), range 2–12 points, respectively. Da Câmara et al. (19) recently reported that an SPPB of ≤ 9 points has 92% sensitivity and 80% specificity for the detection of frailty as well as 81% sensitivity and 52% specificity for Brazilian older adults (19). Considering this cut-off point, ~61.5% of men and women aged 60 years or more in our study population could be classified as frail. According to this definition, around half of the population of 60–69 year olds would be classified as frailty, about three quarters of the population aged 70–79, and the vast majority of octogenarians have a frail (88%) (Figure 1). However, for older adults in an outpatient setting, low agreement has been found between an SPPB score of 7–9 points and prefrailty (0.272), as well as between an SPPB score of 0–6 points and frailty (0.488). For this reason, the implications for diagnostic accuracy can only be interpreted taking into account the pre-test probability and post-test probability. Since no data are available from other Latin American countries with nationally representative, these reference standards could help and guide geriatric medicine as an important tool to assist in the decision-making process regarding physical performance test of lower extremity function in older adults.

Considering these results and the wide use of the SPPB as a recognized physical performance assessment tool for the detection of different health outcomes, the major strength of the present study, is the use of a performance-based physical function assessment that was previously tested for validity and reliability among non-nationally representative Colombian samples (23). Another strength of this study is its focus on the age span of 60 through 96. Finally, an adjusted linear regression analysis was used to display crude descriptive data, and this may bias the presentation and interpretation of the results. This is a very dynamic period in disability transitions and a key age range for the prevention of mobility loss and disability. Nevertheless, some limitations of the present study should be mentioned, including its cross-sectional design, as highlighted above. Thus, a prospective study or trial should be undertaken to confirm the relationship between anthropometric data and physical performance. However, these limitations do not compromise the main findings of this study.

## Conclusion

In summary, this study provides valid national reference standards for older Colombian adults. Because no data are available from South American countries concerning this population, these reference standards could help and guide healthcare professionals in Latin America for physical function classification until their own and/or international reference standards with similar sociodemographic characteristics are available.

## Data Availability Statement

The current study used data from the Ministerio de Salud y la Protección Social de Colombia (https://www.minsalud.gov.co) and legal constraints do not permit public sharing of the data. The Ministerio de Salud y la Protección Social de Colombia, however, is open to all qualified researchers anywhere in the world. Thus, the data used in this communication can be easily and directly accessed by applying through the Ministerio de Salud y la Protección Social de Colombia Management System (https://www.sispro.gov.co/pisis/Pages/pisis-plataforma-de-integraci%C3%B3n-de-SISPRO.aspx).

## Ethics Statement

The studies involving human participants were reviewed and approved by the institutional review boards of the University of Caldas (ID protocol CBCS-021-14) and the University of Valle (ID protocol 09-014 and O11-015). The study protocol for the secondary analysis was approved by the Human Subjects Committee at Pontificia Universidad Javeriana (ID protocol 20/2017-2017/180, FM-CIE-0459-17). The patients/participants provided their written informed consent to participate in this study.

## Author Contributions

LV-S, CC-G, AG-H, FZ-F, MS, and MI: conceptualization. RR-V, LV-S, PH-Q, DR-P, AG-H, and FZ-F: data curation. RR-V, LV-S, CC-G, DR-P, AG-H, and MS: formal analysis. LV-S, CC-G, DR-P, and MI: funding acquisition. RR-V, PH-Q, FZ-F, MS, and MI: investigation. RR-V, MP-S, PH-Q, FZ-F, and MS: methodology. LV-S and CC-G: project administration. CC-G: resources. MP-S: software. RR-V: supervision. RR-V, MP-S, and DR-P: validation. RR-V, MS, and MI: writing—original draft. RR-V and MI: writing—review and editing.

### Conflict of Interest

The authors declare that the research was conducted in the absence of any commercial or financial relationships that could be construed as a potential conflict of interest.
